# Surviving ChatGPT in healthcare

**DOI:** 10.3389/fradi.2023.1224682

**Published:** 2024-02-23

**Authors:** Zhengliang Liu, Lu Zhang, Zihao Wu, Xiaowei Yu, Chao Cao, Haixing Dai, Ninghao Liu, Jun Liu, Wei Liu, Quanzheng Li, Dinggang Shen, Xiang Li, Dajiang Zhu, Tianming Liu

**Affiliations:** ^1^ School of Computing, University of Georgia, Athens, GA, United States; ^2^ Department of Computer Science and Engineering, The University of Texas at Arlington, Arlington, TX, United States; ^3^ Department of Radiology, Second Xiangya Hospital, Changsha, Hunan, China; ^4^ Department of Radiation Oncology, Mayo Clinic, Scottsdale, AZ, United States; ^5^ Department of Radiology, Massachusetts General Hospital and Harvard Medical School, Boston, MA, United States; ^6^ School of Biomedical Engineering, ShanghaiTech University, Shanghai, China; ^7^ Department of Research and Development, Shanhai United Imaging Intelligence Co., Ltd., Shanghai, China; ^8^ Shanghai Clinical Research and Trial Center, Shanghai, China

**Keywords:** ChatGPT, large language models (LLM), Artificial General Intelligence (AGI), GPT-4, Artificial Intelligence-AI

## Abstract

At the dawn of of Artificial General Intelligence (AGI), the emergence of large language models such as ChatGPT show promise in revolutionizing healthcare by improving patient care, expanding medical access, and optimizing clinical processes. However, their integration into healthcare systems requires careful consideration of potential risks, such as inaccurate medical advice, patient privacy violations, the creation of falsified documents or images, overreliance on AGI in medical education, and the perpetuation of biases. It is crucial to implement proper oversight and regulation to address these risks, ensuring the safe and effective incorporation of AGI technologies into healthcare systems. By acknowledging and mitigating these challenges, AGI can be harnessed to enhance patient care, medical knowledge, and healthcare processes, ultimately benefiting society as a whole.

## 1 Introduction

Large language models (LLM) such as ChatGPT and GPT-4 are making significant strides towards the development of Artificial General Intelligence (AGI) (1, 2). Advanced generative AGI holds the potential to improve patient care (3, 4) and streamline healthcare processes (3, 5). However, without proper oversight and regulation, the integration of LLMs into the healthcare system could introduce a range of unintended risks and consequences (4, 6). It is essential to explore these potential risks and address them effectively to ensure that AGI serves as a beneficial aid in the medical field.

One of the foremost concerns regarding AGI in healthcare is the risk of providing inaccurate medical advice (6, 7). Since AI-generated content (AIGC) is based on vast amounts of internet data, there is a possibility that the information provided may be misleading or outright incorrect. For instance, ChatGPT might offer treatment suggestions that are either outdated or not suitable for a specific patient’s condition. Such inaccuracies could result in patients receiving inappropriate treatments or even exacerbate their health issues.

In addition to the significant concern of inaccurate advice and conclusions, the potential violation of patient privacy is another area of concern (8). Although AGI systems like ChatGPT are intended to be securely designed, they may not yet fully comply with privacy regulations like HIPAA (9). Consequently, patient data may be compromised, leading to the unauthorized access, harvesting and sharing of sensitive personal information. This risk highlights the importance of ensuring AGI technologies adhere to strict privacy standards before their widespread adoption in healthcare.

Powerful generative AGI could also be exploited to create fake documents or images, resulting in misinformation and negative consequences (10). For example, unscrupulous individuals or companies might use AGI-generated medical images (11) to support false claims or promote unproven treatments, thereby misleading patients and healthcare professionals.

Moreover, the integration of large language models into medical training could have a detrimental effect on the education of future healthcare professionals (12, 13). Students may rely too heavily on AGI-generated content, neglecting to develop the critical skills required for effective medical practice. By using AGI tools as shortcuts, they might not acquire the ability to differentiate between relevant and irrelevant information, which is crucial in the fast-paced and complex world of healthcare.

Furthermore, established AGI models may inadvertently perpetuate existing biases present in the data used for training (14). Consequently, the outcomes generated by these models might be biased, leading to the reinforcement of stereotypes and potentially causing harm to certain demographics (15). For instance, if a model’s training data contains biased information about a specific ethnic group, the model could produce advice that is detrimental to patients from that group.

In short, while AGI models like ChatGPT hold the potential to revolutionize the healthcare sector, it is essential to recognize and address the potential risks they pose. Proper oversight and regulation are necessary to ensure the integration of AGI technologies into the healthcare system is both safe and effective. By addressing these risks, AGI can be harnessed to significantly improve patient care, medical knowledge, and healthcare processes, ultimately benefiting society as a whole.

**Figure 1:**
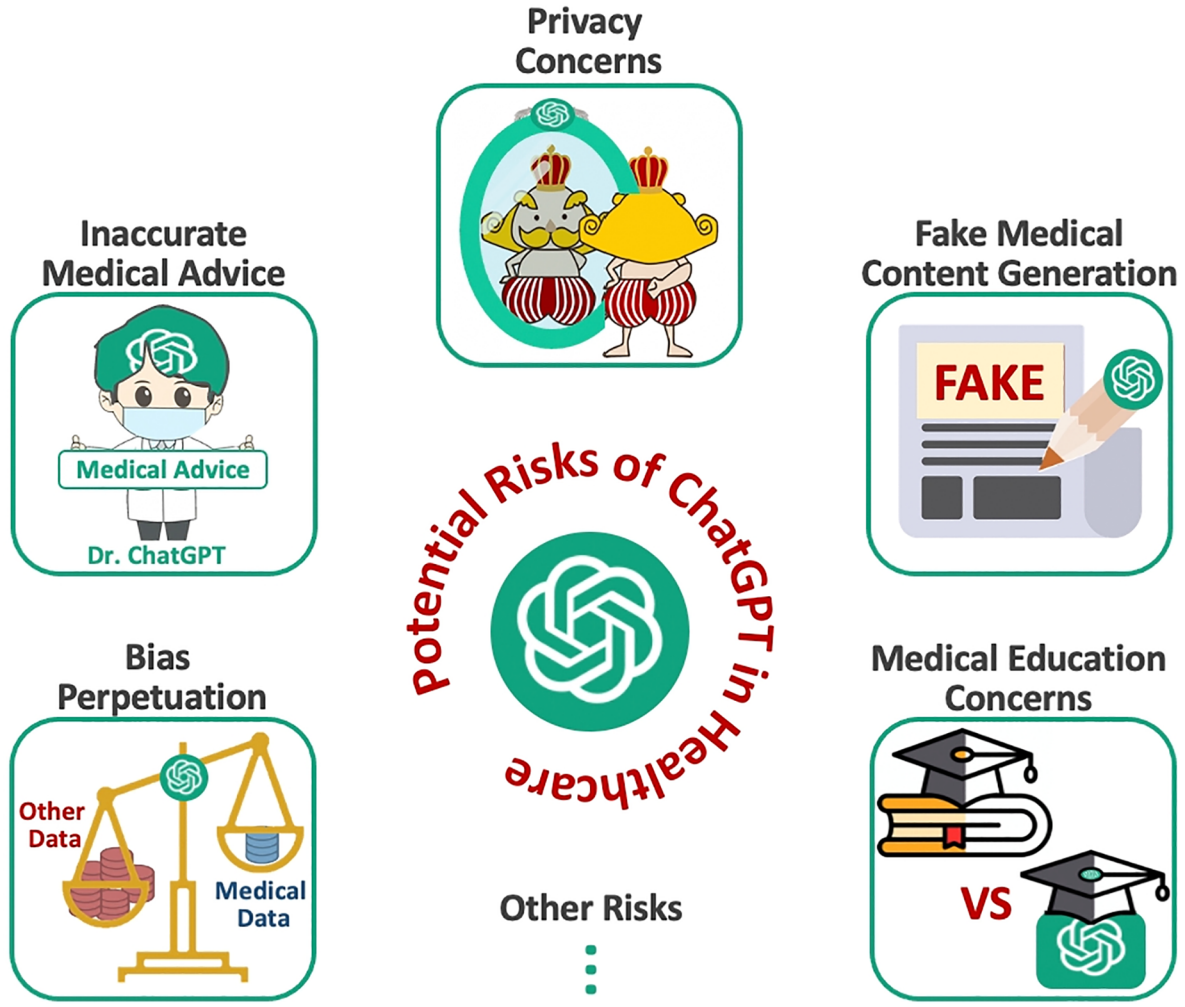
Potential risks of ChatGPT in healthcare.

We divide the ensuing discussion into 6 sections. Each section addresses specific subsets of potential harms and risks posed by large language models such as ChatGPT (see Figure 1 for an overview of this study). We aim to provide a comprehensive yet succinct summary of this subject to spur broader discussion and insights into the future of medicine in the era of AGI. In addition, in Section 7, we discuss the potential risks of AGI models (including ChatGPT) in the context of radiology.

## 2 Truth or dare? Inaccurate medical advice from Dr. ChatGPT

The potential for AGI models, such as ChatGPT, to provide inaccurate medical advice is a critical concern within the medical community (16, 17). As these systems generate content based on vast amounts of data (1, 2, 5), it is possible that the information provided could be misleading or incorrect (18). This raises concerns about the trustworthiness of AGI-generated medical advice, as well as the potential risks to patients who rely on such guidance. This discussion has been ongoing since (19) the introduction of smaller pre-trained models (such as BERT or T5 (20)) to healthcare NLP, but it has become a more prominent concern with the rise of LLMs.

For instance, consider a scenario where a patient consults ChatGPT for recommendations on managing their diabetes. ChatGPT might provide advice based on outdated guidelines, which could lead to inappropriate dietary recommendations or incorrect medication dosages. If the patient were to follow this advice, they might experience adverse effects, such as uncontrolled blood sugar levels or complications arising from improper treatment. For example, in 2018, the American College of Physicians (ACP) issued a new recommendation to control type 2 diabetes patients’ A1C level to between 7% to 8% instead of a previous target of below 7%, since research indicated that reducing the A1C too low through medication did not prevent macrovascular complications yet might lead to substantial harms (21). If ChatGPT or similar models were trained on outdated standards, their responses might lack current medical validity.

Another example concerns the potential for ChatGPT to generate advice that does not consider individual patient factors. Clinical decision-making often requires a nuanced understanding of a patient’s medical history, concurrent health conditions, and potential contraindications. An AGI model like ChatGPT, without direct access to a patient’s medical records, may generate advice that is unsuitable or even harmful to the patient. For instance, a patient suffering from both hypertension and kidney disease may receive medication advice that is appropriate for managing hypertension but exacerbates their kidney condition (22), since ChatGPT is not aware of the full spectrum of the patient’s problems.

Additionally, the rapidly evolving nature of medical knowledge presents challenges for AGI models. With new research findings continuously emerging, it is vital that AGI models are updated regularly to ensure that their advice remains aligned with the latest evidence-based guidelines. However, the lag between the publication of new research and its integration into AGI models may result in patients receiving advice that is no longer considered best practice. Indeed, both ChatGPT and GPT-4 were only trained on data up to September 2021 (23) and consequently have no knowledge of more recent developments.

In the realm of medical diagnostics, the potential for ChatGPT to misinterpret symptoms or overlook critical information could lead to diagnostic errors. For example, a patient may present with symptoms that align with multiple diagnoses. The model might generate advice based on the most common condition, while failing to consider a rare but more serious alternative diagnosis. Such oversight could have serious consequences for the patient, who may not receive the correct treatment in a timely manner.

Overall, there is a significant risk for large language models to produce ungrounded or unverified medical advice. It is necessary to raise awareness of this new challenge in the AGIlandscape. We encourage efforts to instill correct knowledge into models like ChatGPT or establish guardrails that moderate generated medical content.

## 3 The emperor’s new clothes: privacy concerns

Large language models (LLMs) like ChatGPT offer impressive capabilities (24–28), but they also come with significant privacy implications that need to be carefully addressed. One particular risk is the potential for models to inadvertently leak details from the data they were trained on. While this is a concern for all LLMs, there are additional challenges if a model trained on private data (8) were to become publicly accessible.

Datasets used to train language models can be substantial (1, 29), often reaching hundreds of gigabytes, and they draw from various sources and domains (30–34). Consequently, even when trained on public data, these datasets can contain sensitive information, such as personally identifiable information (PII) including names, phone numbers, and addresses. This raises concerns that a model trained on such data could inadvertently expose private details in its output. It is crucial to identify and minimize the risks of such leaks and develop strategies to address these concerns with future models. This has long been a concern in applying language models to healthcare (35). Prior to the advent of LLMs, BERT-based models were typically combined with differential privacy training and federated learning strategies to better protect privacy in healthcare applications (36).

Privacy and data protection regulation compliance is another significant concern associated with LLMs. These models have the capacity to “memorize” personal information, putting it at risk of being discovered by other users or potential attackers (37, 38). This ability to retain and potentially reveal personal information calls for robust measures to ensure data privacy and prevent unauthorized access.

The use of LLMs in healthcare (25, 26) has also raised privacy and security concerns, particularly regarding sensitive medical information (39, 40). Clinical notes, encompassing physician consultations, nursing assessments, lab results, and more, are often stored in free-text formats that may include identifiable or confidential patient information. Unauthorized access to this information poses significant risks to patient confidentiality and privacy. Regulations such as the U.S. Health Insurance Portability and Accountability Act (HIPAA) mandate the removal of re-identifying information from medical records before dissemination to preserve patient confidentiality (41, 42). Researchers are actively exploring ways to mitigate these concerns by employing data masking techniques to conceal sensitive data and prevent unauthorized access (8, 43).

In conclusion, while LLMs offer impressive capabilities, the associated privacy concerns must be diligently addressed. Minimizing data leaks, protecting against unauthorized access, and implementing privacy-preserving techniques are crucial steps toward ensuring the responsible and ethical use of LLMs in safeguarding patient privacy and maintaining data confidentiality.

## 4 All that glitters is not gold: fake medical content generation

Large language models have been increasingly used in fake medical content generation due to their ability to generate text that mimics (10) the writing style and language of medical professionals. These models are based on machine learning algorithms that have been trained on vast amounts of text data, which enables them to generate text that is both coherent and informative. Large language models can also be used to generate fake medical content on a wide range of topics, including diagnosis, treatment, and prognosis. This can be especially useful for medical writers and content creators who need to produce a high volume of content quickly and efficiently.

The use of LLMs for generating fake medical content has become more widespread due to their impressive ability to produce convincing and coherent text that mimics human writing (44). The potential for LLMs, such as ChatGPT, to generate fake medical content raises several ethical concerns, particularly regarding patient safety and informed consent (45, 46). The dissemination of fake medical content may lead to false diagnosis, inappropriate treatments, and further medical complications, causing harm to patients. Additionally, the spread of such fake medical content can have serious public health consequences, as it can fuel the promotion of unproven treatments or products that may be ineffective or even dangerous (16).

To address the risks associated with fake medical content generation using LLMs, several strategies have been proposed (10). One approach is to develop advanced algorithms and tools that can detect fake medical content and prevent its spread. These tools could leverage machine learning and natural language processing techniques to analyze and validate the authenticity of medical content. Another approach is to increase awareness and education among healthcare professionals and the public about the risks associated with fake medical content. By promoting critical thinking and media literacy, individuals can be better equipped to identify fake medical content and make informed decisions about their health.

While LLMs have the potential to transform the content generation of medical data, their misuse for generating fake medical content raises significant ethical concerns. The development of advanced tools to detect and prevent the spread of fake medical content, along with increased awareness and education, can help mitigate the risks associated with the use of LLMs in medical content generation.

## 5 Veritas vos liberabit? AIGC knowledge compromises medical education

The potential misuse of ChatGPT and similar AGI models in medical education raises significant ethical concerns. It is plausible that medical students and trainees could employ these technologies to complete assignments unethically, misrepresenting their actual knowledge and skills through AGI-generated content (AIGC) (47, 48). Such a scenario would not only result in a diminished educational experience for these students but could also jeopardize patient care when they transition into professional practice.

Moreover, relying on AGI tools to generate content might undermine students’ capacity for learning, problem-solving, and generalization from known examples (49, 50). The acquisition of these essential skills is paramount for medical professionals, who must be able to navigate complex situations and adapt to new challenges. If AGI-generated content becomes a crutch for students, they may fail to develop the critical thinking abilities necessary for successful medical practice.

By bypassing the rigorous process of learning and self-discovery, students risk hindering their cognitive growth and reducing their aptitude for medical problem-solving. In the long term, this could lead to a workforce of medical professionals ill-equipped to handle the intricacies of their field, ultimately compromising the quality of healthcare and public trust in the healthcare system.

From another perspective, ChatGPT and similar models offer unique opportunities to democratize medical education that is previously not accessible to the public or medical students in disadvantaged regions. Open medical education offers noticeable benefits in avoiding unwanted treatment (51, 52), making informed decisions (51), promoting effective patient self-management (53) and achieving better clinical outcomes (54). Indeed, ChatGPT enables any audience to quickly source medical information that is previously inaccessible (55), which has a significant social impact, especially for communities and individuals that benefit from the mass dissemination of medical knowledge.

It is crucial to establish guidelines and policies governing the use of AGI models in medical education to mitigate risks and ensure that their potential benefits are harnessed without sacrificing the integrity of the learning process.

## 6 One lie leads to another: bias perpetuation

ChatGPT is a general-purpose LLM that is not specialized for medical problems, even though it can perform better than many models specifically fine-tuned on medical knowledge (56). It is trained on large amounts of real Internet data, which might not be adequately representative of human diversity and could contain pre-existing bias. According to the latest AI Index Report issued by Stanford University, the probability and danger of bias will develop as the size and capabilities of large language models keep growing (57). Previous models, such as BERT, also attracted concerns over bias in their training data (58). Indeed, it is unavoidable for ChatGPT to contain inherent bias. The wide popularity of ChatGPT further exacerbates existing problems.

ChatGPT has shown bias against specific groups since its training data contains racial and sexist stereotypes (59). The issues of underrepresentation, overrepresentation, and misrepresentation in web data are likely introducing various kinds of bias into ChatGPT. The outputs containing bias typically manifest in nuanced representations, which makes it difficult to recognize and correct bias and toxicity (60–62).

Also, fine-tuning ChatGPT on historical medical data has the potential to introduce or exacerbate bias that already exists within the data. For example, clinical practice biases, such as under-testing of marginalized communities, can impact the underlying clinical data and introduce bias during future training (63).

In addition, implicit bias from healthcare professionals can manifest in clinical notes, including segments of diagnoses and treatment decisions (64). ChatGPT and GPT-4 might introduce these new biases into downstream applications if such notes are used for training.

OpenAI has released plugins for images and will certainly develop multimodal foundational models in the future (e.g., GPT-4 will have capabilities to process images in the near future). But fairness research indicates that the combination with additional information or modality may not necessarily improve performance and is likely to bring about new unfairness and bias (65). For example, in CLIP, a language-vision model, historical race and gender bias are reinforced (66).

Fostering research and development to detect, mitigate, and prevent bias, toxicity, and other undesirable behaviors in large language models like ChatGPT and GPT-4 are crucial for a responsible AI future. By actively pursuing these objectives, we can ensure that these powerful tools serve as inclusive, unbiased, and beneficial resources for users across diverse backgrounds. This pursuit not only safeguards the ethical foundations of AI but also greatly enhances its potential to positively impact society.

## 7 Potential risks of artificial general intelligence (AGI) models in radiology

*Inaccurate medical advice*: A substantial risk associated with AGI models like ChatGPT involves the provision of inaccurate medical advice, particularly in the nuanced field of radiology. For instance, when interpreting a radiology report indicating the presence of a small pulmonary nodule, the model might suggest watchful waiting based on outdated guidelines, overlooking recent research that indicates a higher malignancy risk requiring more active intervention.

*Privacy Concerns*: Privacy is a paramount concern in the use of AGI models. For example, patients seeking guidance for understanding a radiology report could unwittingly disclose sensitive health data like a past diagnosis of breast cancer or a family history of genetic disorders. Potential security vulnerabilities in the wider AGI system could expose this sensitive data to misuse or unauthorized access.

*Fake Medical Content Generation*: The potential for AGI models to generate misleading or false medical content presents a significant risk. An individual with malevolent intent could misuse AGI tools to fabricate radiology reports, falsely indicating the presence or absence of a medical condition, such as fabricating a report showing a clean bill of health when the actual scan revealed lung nodules indicative of early-stage cancer.

*Compromise of Medical Education*: AGI models have the potential to unintentionally undermine the quality of medical education. For example, a medical trainee could become overly reliant on a multimodal AGI model for interpreting brain MRI scans for assignments. This over-reliance could deprive them of crucial learning experiences and inadvertently foster an environment of plagiarism and excessive dependence on automation. Consequently, this may lead to the undesired outcome of inadequately trained professionals tasked with handling complex or ambiguous clinical situations.

*Bias Perpetuation*: Lastly, AGI models can unintentionally propagate existing biases in medical data. For example, if the data used to train ChatGPT and GPT-4 over-represents Caucasian individuals, the models might be less adept at interpreting radiology reports concerning conditions more prevalent in other ethnic groups, such as the higher incidence of sarcoidosis in African American populations (67).

## 8 Conclusion

In conclusion, while the emergence of large language models such as ChatGPT offers promising prospects for revolutionizing healthcare, addressing the potential risks and challenges is of paramount importance. Future research should focus on developing robust methods to ensure the accuracy, reliability, and privacy compliance of medical contents generated by AGI, as well as monitoring and mitigating the biases that may be introduced during the training of these models. Additionally, guidelines and regulations should be established to govern the use of AGI models in medical education, promoting their responsible use and preserving the integrity of the learning process.

By acknowledging and addressing these challenges, Artificial General Intelligence can be harnessed to revolutionize patient care and healthcare, ultimately benefiting society as a whole and significantly promoting national health. The interdisciplinary collaboration between AGI researchers, medical professionals, ethicists, and policy-makers will play a crucial role in shaping the future of medicine in the era of AGI, ensuring its safe and effective integration into healthcare systems worldwide.
